# *Toxoplasma gondii* seroprevalence among pregnant women in Africa: A systematic review and meta-analysis

**DOI:** 10.1371/journal.pntd.0012198

**Published:** 2024-05-23

**Authors:** Yared Mulu Gelaw, Gizachew Worku Dagnew, Getu Degu Alene, Jean-Pierre Gangneux, Florence Robert-Gangneux

**Affiliations:** 1 School of Public Health, College of Medicine and Health Sciences, Bahir Dar University, Bahir Dar, Ethiopia; 2 Irset (Institut de Recherche en Santé, Environnement et Travail), UMR_S 1085, Université de Rennes, CHU Rennes, Inserm, EHESP, Rennes, France; University of Strathclyde, UNITED KINGDOM

## Abstract

**Background:**

Toxoplasmosis is a serious endemic zoonotic disease caused by the protozoan parasite *Toxoplasma gondii*. *Toxoplasma* infection during pregnancy can result in congenital transmission and serious fetal and neonatal complications. This systematic review and meta-analysis aimed to assess the pooled seroprevalence of *T*. *gondii* infection and its determinants among pregnant women in African countries.

**Methods:**

All articles reporting the seroprevalence of toxoplasmosis among pregnant women in African countries and published from 2010 to 2023 were searched using various databases. The pooled prevalence of toxoplasmosis was calculated using a random-effect model. The variation between the included studies was assessed using a funnel plot and I^2^ heterogeneity statistics. To identify the sources of heterogeneity, sub-group analysis was further conducted by country, diagnostic method, and sub-African region. The association of prevalence rates with the socio-economic level and geoclimatic parameters was also explored.

**Results:**

In total, 29,383 pregnant women from 60 articles were included for analysis. The pooled *T*. *gondii* seroprevalence was 42.89% with high heterogeneity (I^2^ = 99.4%, P < 0.001). Sub-group analysis revealed variation by country (ranging from 2.62% in Namibia to 80.28% in Congo), diagnostic method used (from 8.66% in studies using a rapid diagnostic test to 55.69% in those using an agglutination test), and sub-African region (from 4.14% in regions of Southern Africa to 53.96 in Central Africa). Cat ownership (OR = 1.58) and the consumption of raw meat (OR = 1.50) and raw vegetables (OR = 1.48) had a statistically significant combined effect on *T*. *gondii* seroprevalence. No association was found between *T*. *gondii* prevalence and the level of income of the country or geoclimatic parameters.

**Conclusion:**

The prevalence of toxoplasmosis infection among pregnant women in Africa is high, particularly in Central and Eastern Africa. The determinants of prevalence are multifactorial. Therefore, efforts should be made to increase the awareness of women concerning the risk factors for toxoplasmosis.

## Author summary

*Toxoplasma gondii* is a zoonotic protozoan parasite that infects both humans and animals. *T*. *gondii* infection during pregnancy causes congenital transmission and serious fetal and neonatal complications. Estimating the pooled prevalence of toxoplasmosis in high-risk population groups could catch the attention of policymakers and prompt them to develop a policy brief and guidelines to reduce toxoplasmosis sequelae. In this systematic review and meta-analysis, we aimed to assess the pooled seroprevalence of *T*. *gondii* infection and its determinants among pregnant women in Africa. All articles reporting recent data on the seroprevalence of toxoplasmosis among pregnant women in African countries were systematically searched using various databases. Sixty articles published from 2010 to 2023 were included for analysis, representing a total of 29,383 pregnant women. The pooled prevalence of toxoplasmosis was 42.89% (95%CI: 36.75–49.03). Cat ownership and the consumption of raw meat and raw vegetables had a statistically significant effect on the seroprevalence of toxoplasmosis among pregnant women in Africa. The infection is more prevalent in countries of Central and East Africa. These findings should increase the awareness of health workers and their understanding of toxoplasmosis among pregnant women and motivate them to conduct integrated community awareness creation activities concerning the risk factors for toxoplasmosis.

## Introduction

Toxoplasmosis is an endemic zoonotic disease caused by *Toxoplasma gondii*, a protozoan parasite with a worldwide distribution [1,2]. Cats and other felids are the definitive hosts of the parasite and humans, as well as virtually all warm-blooded animals, are intermediate hosts. They are contaminated by the ingestion of food or water containing oocysts spread in the environment through cat feces [2]. Intermediate hosts undergo a systemic infection that can be controlled by an efficient Th1 immune response, but not eradicated, leading to the lifelong persistence of encysted parasites, mainly in the muscles, brain, and eyes. Therefore, the infection can also be acquired through the ingestion of contaminated meat if eaten undercooked [2–4]. When primary infection occurs in a pregnant women, *T*. *gondii* can be transmitted through the placental barrier, resulting in congenital infection of varying severity, depending on the stage of gestation, or loss of the fetus [2,5].

An estimated one-third of the world population is infected with *T*. *gondii*, which is responsible for 1.2 million disability-adjusted life years (DALYs) each year [6]. The seroprevalence of toxoplasmosis among pregnant women varies greatly between geographical areas as a result of combined factors, including the climate, socio-economic level and standard of living, quality of the drinking water, and proximity with cats or other felids. There is a trend towards a decreasing prevalence in European countries, such as France [7], likely related to modifications in dietary habits or food hygiene or handling. In Africa, a previous meta-analysis estimated that more than half of African pregnant women are chronically infected with *Toxoplasma* [8] but the selection criteria and inclusion period were not clear. Several studies from African countries reported high variability in *T*. *gondii* seroprevalence among pregnant women, ranging from below 5 to 80% [9,10]. In a recent Egyptian review, Abbas et al. [11] reported a seroprevalence ranging from 4 to 71% among pregnant women, depending on the geographical location. Interestingly, it was as high as 27 to 100% among women with a history of recent abortion, suggesting that the burden of toxoplasmosis could be higher than suspected. In addition, epidemiological studies have shown that 95% of cats are seropositive for *T*. *gondii*, suggesting massive contamination of the environment with oocysts.

Although there are limited country- and sub-continent-level data about *T*. *gondii* seroprevalence among pregnant women in Africa, there have been no studies to assess its predictors across this continent. Knowing the seroprevalence and predictors of *T*. *gondii* infection could help health authorities to have a better understanding of the level of risk according to local factors and design targeted intervention measures to reduce fetal sequelae related to the infection. This systematic review and meta-analysis aimed to assess the seroprevalence of *Toxoplasma gondii* infection and its predictors among pregnant women in Africa.

## Methods and materials

### Study protocol and eligibility

This systematic review and meta-analysis is reported using the preferred Reporting Items for Systematic Review and Meta-analysis (PRISMA) reporting checklist.

All cross-sectional and cohort observational studies conducted in various countries of Africa on the prevalence of toxoplasmosis among pregnant women published in the English language from January 1, 2010, to March 30, 2023, were included.

### Sources of information and search strategies

We searched through various databases to retrieve pertinent findings about the prevalence of toxoplasmosis infection among pregnant women (PubMed, Researchgate, Africa Journal Online (AJOL), and Europe PMC using the following Mesh terms (“Toxoplasmosis” OR “Toxoplasma gondii”), AND ("Pregnant Women” OR “Prenatal care”), AND (“Africa”) or combining with every individual African country (S1 File).

### Study selection, data extraction, and quality assurance

Duplicated articles were removed using Endnote X8 citation manager. Initially, all retrieved articles were screened against the eligibility criteria via careful reading of the article title and abstract using the CoCoPoP (Condition, Context, and Population) framework. The condition (Co) was the prevalence of *T*. *gondii* and was the main outcome of interest, the study population (Pop) was pregnant women, and the context (Co) was a study conducted in all African countries. Articles fulfilling the inclusion criteria were included and reviewed. The eligibility of each study was assessed by two independent investigators. The inter-rater agreement between the two reviewers was rated using the “kappa index” tool [12] and the result showed almost perfect agreement between them (observed agreement (Po) = 0.94, kappa cofficient (k) = 0.832) (S2 File). Discrepancies between the two investigators were resolved by consensus after the paper was reviewed by a third reviewer.

The reviewers independently collected data from all eligible publications using a standardized form. The following data were captured and recorded using Microsoft Excel 2016: author, year of publication, country, study subject, study design, sample population, number of cases (participants with an outcome), diagnostic tool/method, and prevalence. The quality of the included studies and the risk of bias were critically evaluated using the revised Joanna Briggs Institute (JBI) eight-item critical appraisal tool to assess the quality of observational studies (analytical cross-sectional studies) [13] (S3 File). The quality ranking of each full extracted article was scored as low (≤ 3 of 8), medium (between 3 and 6 of 8), or high (≥ 6 of 8). To reduce the risk of bias, we only included articles with higher quality assessment scores.

### Strategy of data analysis

Data were imported to Stata version 15.1 from a Microsoft Excel sheet for analysis. The pooled prevalence of toxoplasmosis, with the 95% confidence interval (95%CI), was determined using a random effect model. Heterogeneity between the included studies was examined using I^2^ statistics. Heterogeneity is considered when the p-value of I^2^ is < 0.05. I^2^ statistics results of 25, 50, and 75% represent low, moderate, and high heterogeneity, respectively. An Egger regression asymmetry test for meta-analysis was used to assess publication bias. An Egger test p-value < 0.05 shows the presence of publication bias. Subgroup analysis and meta-regression were performed to verify the sources of heterogeneity. Moreover, sensitivity analysis was carried out to ensure the robustness of the result and to determine the influence of a single study. To assess the influence of a single study, the pooled prevalence was calculated leaving one study out at a time.

Data on potential predictor variables such as residence, cat ownership, the consumption of raw meat or raw vegetables, the drinking of raw milk, contact with soil, a history of blood transfusion, and HIV status were recorded and analyzed.

## Results

**Fig 1:**
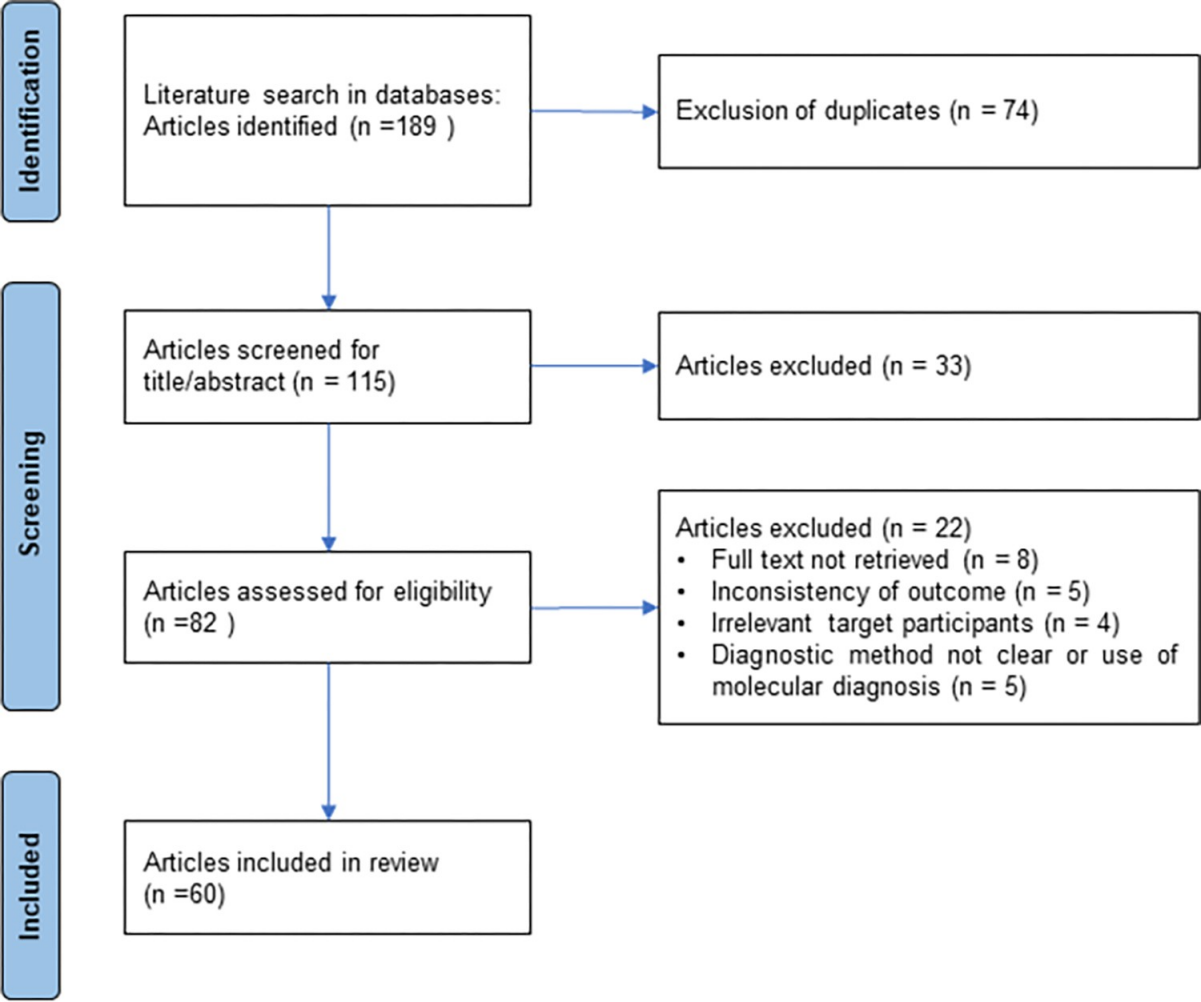
PRISMA flow diagram of the studies included in the systematic review and meta-analysis using the updated 2020 PRISMA flow diagram.

In total, 189 articles were initially identified from various electronic databases, of which 74 were removed due to duplication. After screening using the article title and abstract, 82 articles were selected for full retrieval. Twenty-two were further excluded due to the inability to retrieve the full text of the article, the use of a language other than English, or an inadequate target population, outcome, or diagnostic method. Finally, 60 articles were included for analysis (Fig 1).

### Characteristics of included studies

In total, 60 full document articles published between January 1, 2010, and March 30, 2023, from 19 different African countries were included in this systematic review and meta-analysis [10,14–73]. Approximately half (48.4%) of the included studies were from three countries: Ethiopia (14 studies), Nigeria (9 studies), and Tanzania (6 studies). In terms of the sub-regions of Africa, most of the included articles were from East Africa (28 studies) or West Africa (17 studies). In total, 29,383 pregnant women were included. An average of 490 pregnant women were included in each study, ranging from 90 in a Nigerian study [64] to 5,578 in one from Morocco [21]. Most studies (87.1%) used a cross-sectional study design. The presence of anti-*Toxoplasma gondii* antibodies was assessed by enzyme-immunoassay (EIA) (48 studies), agglutination tests (AT) (10 studies), or rapid diagnostic tests (RDTs) (2 studies) (Table 1).

**Table 1:** Characteristics of the 60 studies included in the systematic review and meta-analysis to estimate the seroprevalence of toxoplasmosis among pregnant women in Africa.

First author, year	Reference no	Country	Study design	Sample size	Number of seropositive women	Prevalence (%)	Diagnostic tool	Quality score
Hassan SA et al., 2023	14	Somalia	CS	403	182	45.16	EIA	6
Okojokwu O et al., 2023	15	Nigeria	CS	158	44	27.8	EIA	7
Dambrun M et al., 2022	16	Benin	RS	974	512	52.56	EIA	8
Lushina M et al., 2022	17	Tanzania	CS	383	104	27.15	EIA	8
Laboudi M et al., 2021	18	Morocco	RS	637	274	43	EIA	6
Adugna B et al., 2021	19	Ethiopia	CS	401	284	70.82	AT	6
Singh B et al., 2021	20	Ghana	CS	400	229	57.25	EIA	8
Hoummadi L et al., 2020	21	Morocco	RS	5578	1611	28.88	EIA	6
Khames M et al., 2020	72	Algeria	CS	1012	252	24.9	EIA	6
Akubuilo AS et al., 2020	22	Nigeria	CS	384	45	11.72	RDT	6
Van der Colf BE et al., 2020	9	Namibia	CS	344	9	2.62	EIA	7
Zakari, M et al., 2020	24	Nigeria	CS	320	92	28.75	EIA	7
Mulugeta et al., 2020	25	Ethiopia	CS	233	158	67.81	AT	8
Tecle AS et al., 2020	26	Eritrea	CS	210	112	53.33	EIA	6
Nguemaïm NF et al., 2020	27	Cameroon	CS	127	44	34.65	EIA	7
Abdelbaset AE et al., 2020	28	Egypt	CS	96	22	22.92	EIA	6
Fenta., 2019	29	Ethiopia	CS	500	409	81.8	EIA	8
Teweldemedihin et al., 2019	30	Ethiopia	CS	360	128	35.56	EIA	8
Mirambo MM.et al., 2019	31	Tanzania	CS	300	92	30.67	EIA	7
Todjom FG et al., 2019	32	Cameroon	CS	200	91	45.5	EIA	7
Mabeku LB et al., 2018	33	Cameroon	CS	643	230	35.77	EIA	7
Jula et al., 2018	34	Ethiopia	CS	401	96	23.94	EIA	7
Adeniyi OT et al., 2018	35	Nigeria	CS	391	119	30.43	EIA	7
Hafez Hassanain et al., 2018	73	Egypt	CS	388	79	20.36	EIA	6
Dairo MD et al., 2018	37	Nigeria	CS	377	135	35.81	EIA	8
Mohammed WM et al., 2018	38	Sudan	CS	300	68	22.67	EIA	6
Paul et al., 2018	39	Tanzania	CS	254	113	44.49	EIA	7
Tlamcani Z et al., 2017	40	Morocco	PS	3440	1367	39.74	EIA	6
Pegha Moukandja et al., 2017	41	Gabon	RS	973	557	57.25	EIA	6
El-Shqanqery HE et al., 2017	42	Egypt	CS	693	209	30.16	EIA	6
Frimpong et al., 2017	43	Zambia	CS	411	24	5.84	RDT	8
Ballah F et al., 2017	44	Nigeria	CS	400	112	28	EIA	7
Murebwayire E et al., 2017	45	Rwanda	CS	384	47	12.24	EIA	8
Saajan AM et al., 2017	46	Tanzania	CS	347	154	44.38	EIA	6
Bamba et al., 2017	47	Burkina Faso	CS	316	98	31	AT	8
Negussie A et al., 2017	48	Ethiopia	CS	301	105	34.88	AT	7
Yohanes T et al., 2017	49	Ethiopia	CS	232	184	79.31	EIA	8
Negero et al., 2017	50	Ethiopia	CS	210	159	75.71	AT	8
Völker et al., 2017	51	Ghana	CS	168	123	73.21	EIA	7
Oboro IL et al., 2016	52	Nigeria	CS	288	189	65.62	EIA	6
Abamecha et al., 2016	53	Ethiopia	CS	232	198	85.34	EIA	8
Ayi I et al., 2016	54	Ghana	CS	125	64	51.2	EIA	6
Kwofie KD et al., 2016	55	Ghana	CS	100	38	38	EIA	6
Awoke K et al., 2015	56	Ethiopia	CS	384	71	18.49	AT	8
Nasir IA et al., 2015	57	Nigeria	CS	360	176	48.89	EIA	6
Gelaye et al., 2015	10	Ethiopia	CS	288	246	85.42	AT	8
Agmas et al., 2015	58	Ethiopia	CS	263	180	68.44	AT	8
Elichilia R Shao et al., 2015	59	Tanzania	CS	144	60	41.67	EIA	8
Doudou et al., 2014	60	Congo	CS	781	627	80.28	EIA	6
Endris M et al., 2014	61	Ethiopia	CS	385	341	88.57	AT	8
Bamba S et al., 2014	62	Burkina Faso	CS	348	121	34.77	EIA	6
Abdel-Raouff M et al., 2014	63	Sudan	CS	163	33	20.25	EIA	6
Oyinloye SO et al., 2014	64	Nigeria	CS	90	20	22.22	EIA	6
Mwambe et al., 2013	65	Tanzania	CS	350	108	30.86	EIA	8
EI Deeb et al., 2012	66	Egypt	CS	323	218	67.49	EIA	6
Zemene et al., 2012	67	Ethiopia	CS	201	168	83.58	EIA	7
Linguissi et al., 2012	68	Burkina Faso	RS	182	37	20.33	EIA	6
Sattti, A.B et al., 2011	69	Sudan	PS	455	72	15.82	AT	6
Njunda AL et al., 2011	70	Cameroon	CS	110	77	70	EIA	7
Sitoe et al., 2010	71	Mozambique	CS	150	28	18.67	EIA	6

CS: cross-cectional ctudy, RS: retrospective study, PS: prospective study EIA: enzyme immunoassay, AT: agglutination test, RDT: rapid diagnostic test

### Toxoplasma seroprevalence among pregnant women in Africa

**Fig 2:**
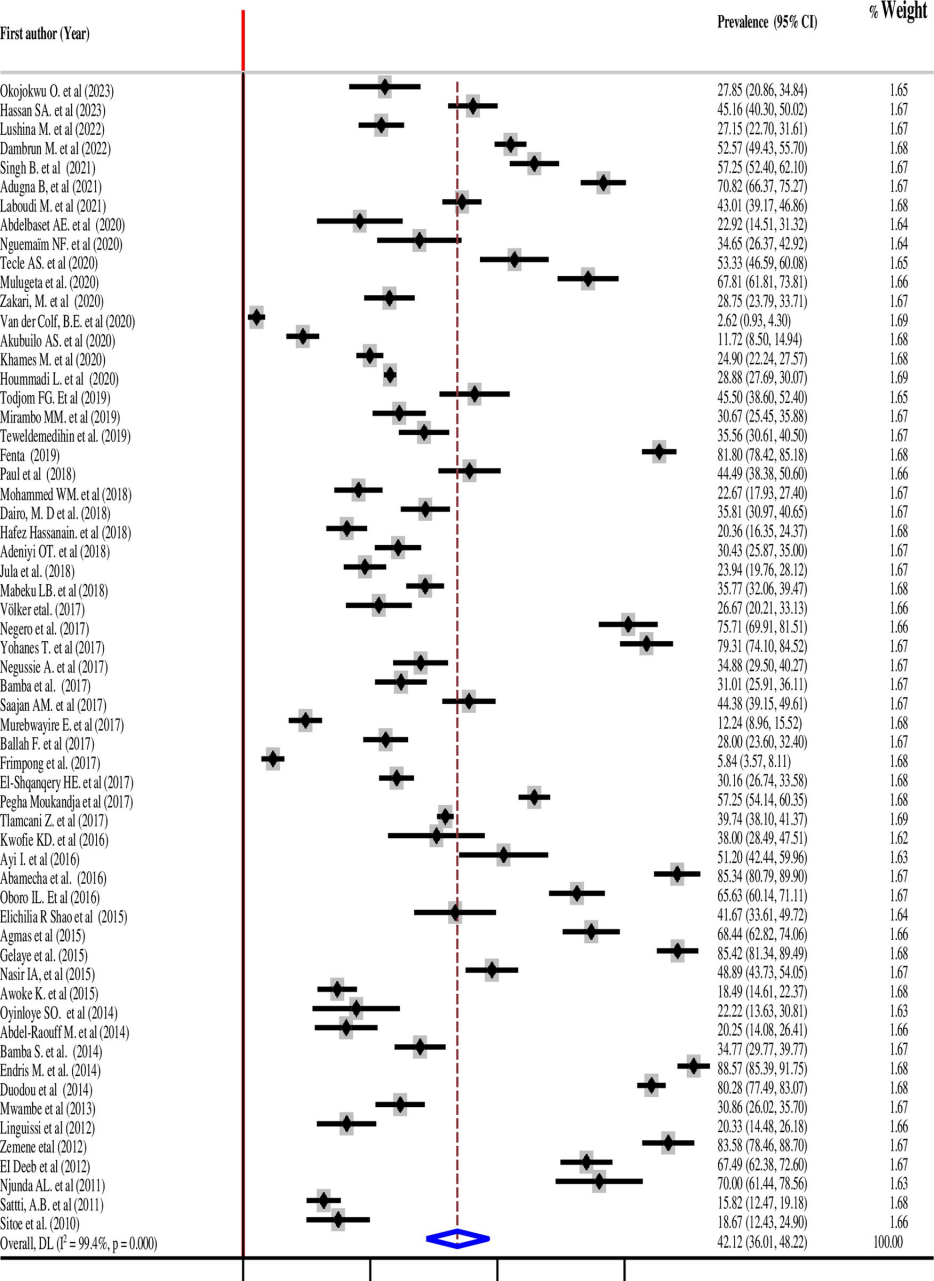
Pooled prevalence of toxoplasmosis among pregnant women in Africa (n = 60 articles included, references #9,10, 14–73).

Across the 60 studies included in this systematic review, the prevalence of toxoplasmosis in pregnant women ranged from 2.61% in Namibia [9] to 88.6% in Ethiopia [61]. The pooled prevalence of toxoplasmosis was 42.12% (95%CI: 36.01–48.22). I^2^ statistics showed the presence of heterogeneity (I^2^ = 99.3%, 95%CI: 98.7% - 99.6%, P < 0.001) (Fig 2). Cochran’s Q statistic was 9127.26, with a p-value < 0.001, and the test of overall effect was 0 (z = 13.524, p < 0.001). Subgroup analysis was thus carried out to identify the sources of heterogeneity.

### Publication bias

**Fig 3:**
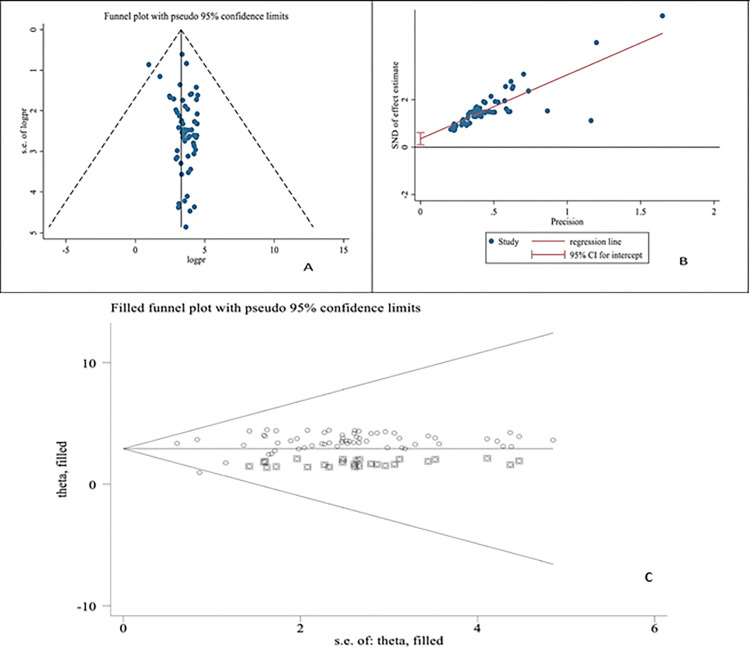
Funnel plot (A), Egger publication bias plot (B) of the logit event rate, and filled funnel plot of the logit event rate (C) of toxoplasmosis among pregnant women in Africa.

The funnel plot and bias coefficient (Egger’s test; b = 35.2%, 95%CI: 9.6–60.9, P = 0.008) for studies published on *T*. *gondii* seroprevalence among pregnant women in Africa confirmed the presence of publication bias and a small-study effect. The results of trim analysis also revealed the presence of publication bias (Q = 14.162 on 61 degrees of freedom (p = 1.000) and Moment-based estimate of between-study variance = 0.000), with a pooled estimate of 3.299, 95%CI: 2.826–3.773, p-value < 0.001) (Fig 3).

### Sub-group analysis

Sub-group analysis was conducted using the following variables: country, sub-African region, publication period, serological method, and type of study design. Sub-group analysis by country showed the highest mean seroprevalence to be observed in Congo (80.28%), followed by 64.28% in Ethiopia, whereas the lowest was 2.62% in Namibia and 5.84% in Zambia (Table 2). The results of country-level I^2^ statistics showed the presence of heterogeneity between studies of the same country, except for the studies included in Sudan (I^2^ = 65.1%, P = 0.057). On the other hand, the sub-group analysis by Sub-African region showed the highest prevalence of toxoplasmosis to be in Central Africa (53.96%, 95%CI: 37.30–70.63) and the lowest in the South African region (4.14%, 95%CI: 0.98–7.29). As indicated by overlapping of the 95%CI, there was a significant difference between the South African region and other sub-African regions (Table 2).

**Table 2:** Sub-group analysis for the seroprevalence of toxoplasmosis among pregnant women in Africa.

Sub-group	No. of included studies	Total sample size	Prevalence (95%CI)	Heterogeinity statistics		
				Cochran’s Q	I^2^	P-value
**By Country**						0.000
Somalia	1	403	45.16 (40.30–50.02)	0.00	- - - -	- - - -
Nigeria	09	2768	33.27 (22.42–44.11)	347.52	97.7%	0.000
Benin	1	974	52.57 (49.43–55.70)	0.00	- - - -	- - - -
Tanzania	06	1778	36.30 (29.87–42.72)	41.63	88.0%	0.000
Morocco	03	9655	37.10 (28.23–45.97)	137.08	98.5%	0.000
Ethiopia	14	4391	64.26 (50.35–78.18)	1758.89	99.3%	0.000
Ghana	04	793	55.26 (42.37–68.15)	39.21	92.3%	0.000
Eritrea	01	210	53.33 (46.59–60.08)	0.00	- - - -	- - - -
Namibia	01	344	2.62 (0.93–4.30)	0.00	- - - -	- - - -
Algeria	01	1012	24.90 (22.24–27.57)	0.00	- - - -	- - - -
Cameroon	04	1080	46.25 (32.25–60.24)	55.60	94.6%	0.000
Egypt	04	1500	35.28 (15.07–55.49)	221.13	98.6	0.000
Sudan	03	918	19.26 (14.72–23.80)	5.72	65.1%	0.057
Burkina Faso	03	846	28.83 (20.71–36.95)	14.05	85.8%	0.001
Gabon	01	973	57.25 (54.14–60.35)	0.00	- - - -	- - - -
Zambia	01	411	5.84 (3.57–8.11)	0.00	- - - -	- - - -
Rwanda	01	384	12.24 (8.96–15.52)	0.00	- - - -	- - - -
Congo	01	781	80.28 (77.49–83.07)	0.00	- - - -	- - - -
Mozambique	01	150	18.67 (12.43–24.90)	0.00	- - - -	- - - -
**By Sub-African Region**						
East Africa	27	9234	48.41 (37.50–59.32)	3859.33	99.3%	0.000
West Africa	17	5381	38.66 (30.32–47.01)	736.5	97.8%	0.000
North Africa	08	12167	34.72 (27.74–41.71)	393.12	98.2%	0.000
Central Africa	06	2831	53.96 (37.30–70.63)	428.52	98.8%	0.000
South Africa	02	755	4.14 (0.98–7.29)	5.00	80.0%	0.025
**By diagnostic method**						0.000
EIA	48	25340	41.65 (35.51–47.79)	5851.67	99.2%	0.000
AT	10	3236	55.69 (36.07–75.31)	1781.96	99.5%	0.000
RDT	02	795	8.66 (2.91–14.42)	8.57	88.3%	0.003
**By type of study design**						0.000
Cross-sectional study	53	17132	43.70 (36.01–51.39)	8594.62	99.4%	0.000
Retrospective study	05	8344	40.49 (26.71–54.26)	459.76	99.1%	0.000
Prospective study	02	3895	27.83 (4.39–51.26)	157.82	99.4%	0.000

EIA: enzyme immunoassay, AT: agglutination test, RDT: rapid diagnostic test

### Sensitivity analysis

We further investigated the sources of heterogeneity in the pooled *T*. *gondii* seroprevalence in Africa and ensured the robustness of the data by performing a sensitivity analysis. The removal of each study one by one and subsequent calculation of the pooled prevalence showed the seroprevalence of toxoplasmosis among pregnant women to vary between 42.11% (95%CI: 36.20–48.01) following the removal of the study of Endris et al. (2014) [61] and 43.58% (95%CI: 37.73, 49.42) following the removal of that of Van der Colf et al. (2020) [9]. Based on this analysis, we concluded that our estimation of the pooled prevalence was robust and showed no evidence in favor of a single-study influence (S4 File).

**Fig 4:**
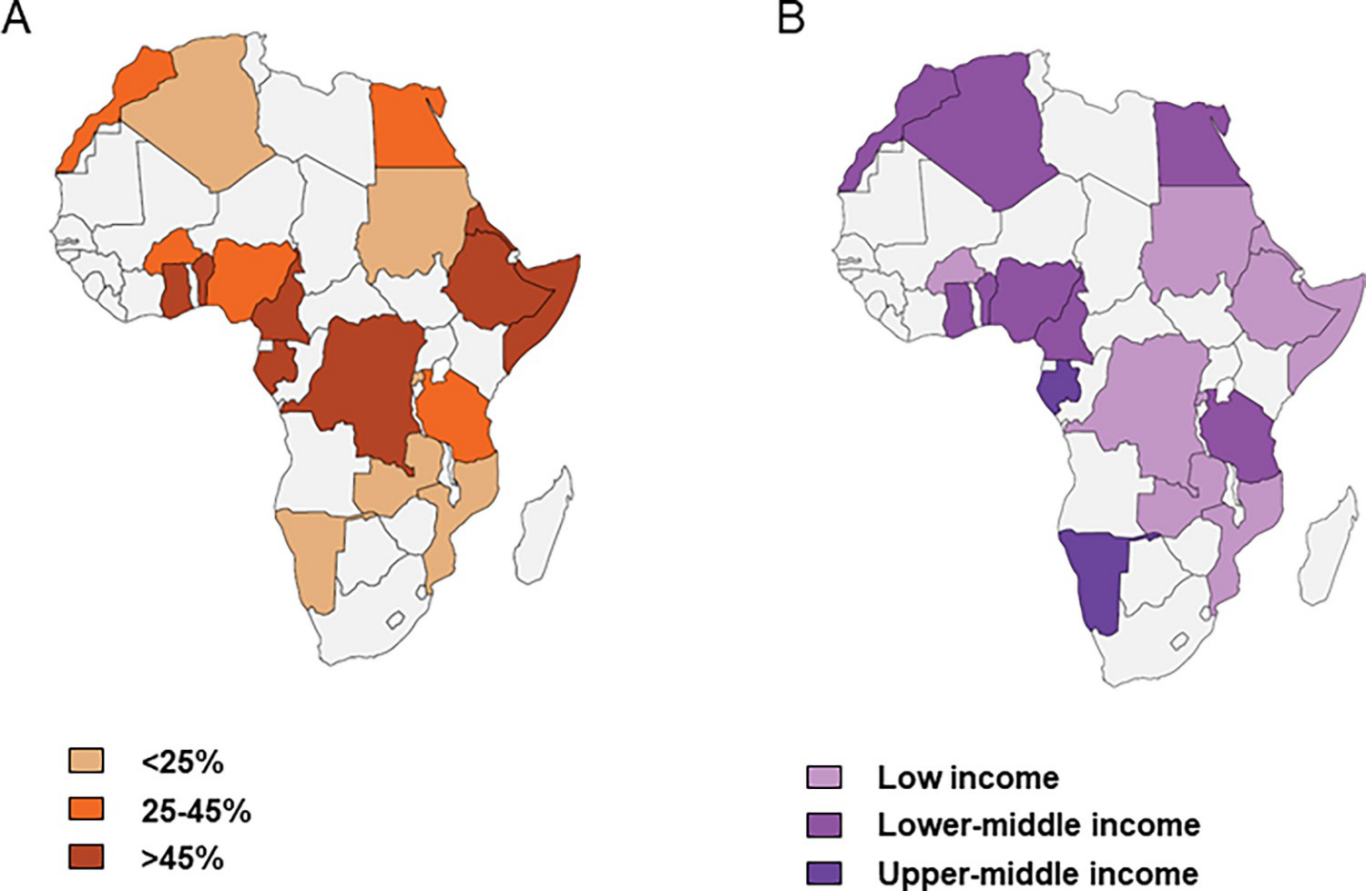
**Toxoplasma seroprevalence among pregnant women in different African countries (A) and the income level of the countries with an indicated seroprevalence (B).** Maps were built using the free website MapChart (https://www.mapchart.net/index.html).

Of the 19 countries with documented seroprevalence among pregnant women from 2010 onwards, six had a seroprevalendce of < 25%, five of 25% to 45%, and eight of > 45% (Table 3 and Fig 4). Half of the countries with a *Toxoplasma* prevalence > 45% were low-income countries (Table 3). However, 4/6 countries with a seroprevalence < 25%, were also low-income countries, showing that poverty cannot solely explain the risk of *Toxoplasma* exposure.

**Table 3:** Characteristics of the 19 African countries (60 articles included).

Country	Prevalence (%, 95%CI)	African geographical area	Average elevation (m)	Annual average precipitation (mm)	Climate	GDP per capita (USD, 2022) ^a^	Income group (World Bank)
Somalia	45.16 (40.30–50.02)	East	410	282	Arid	470	Low
Nigeria	33.27 (22.42–44.11)	West	380	1150	Mixt	2140	Lower- middle
Benin	52.57 (49.43–55.70)	West	273	1039	Tropical	1400	Lower -middle
Tanzania	36.30 (29.87–42.72)	East	1018	1071	Tropical	1200	Lower -middle
Morocco	37.10 (28.23–45.97)	North	909	346	Mixt	3710	Lower- middle
Ethiopia	64.26 (50.35–78.18)	East	1330	848	Tropical	1020	Low
Ghana	55.26 (42.37–68.15)	West	190	1187	Tropical	2350	Lower -middle
Eritrea	53.33 (46.59–60.08)	East	853	384	Mixt	646	Low
Namibia	2.62 (0.93–4.30)	South	1141	285	Arid	4880	Upper- middle
Algeria	24.90 (22.24–27.57)	North	800	89	Mixt	3900	Lower -middle
Cameroon	46.25 (32.25–60.24)	West	667	1604	Tropical	1660	Lower -middle
Egypt	35.28 (15.07–55.49)	North	321	18	Arid	4100	Lower -middle
Sudan	19.26 (14.72–23.80)	East	568	250	Arid	760	Low
Burkina Faso	28.83 (20.71–36.95)	West	297	748	Arid	840	Low
Gabon	57.25 (54.14–60.35)	Central	377	1831	Tropical	7540	Upper-middle
Zambia	5.84 (3.57–8.11)	Central	1138	1020	Tropical	1170	Low
Rwanda	12.24 (8.96–15.52)	East	1598	1212	Tropical	930	Low
Democratic Republic of Congo	80.28 (77.49–83.07)	Central	726	1543	Tropical	590	Low
Mozambique	18.67 (12.43–24.90)	South	345	1032	Tropical	500	Low

^a^ Income level of African country (World Bank 2022–2023): low ≤ 1,085 USD; low-middle = 1,086–4,255 USD; opper middle income = 4,256–13,205, high ≥ 13,205

We found no association between *Toxoplasma* seroprevalence and geographical features, such as the type of climate, average elevation, or annual rainfall observed in the countries (Table 4).

**Table 4:** Prevalence according to income level and geographical characteristics.

	No of countries with indicated prevalence			p-value
	< 25%	25–45%	> 45%	
Income level (GDP per capita)				0.318
Low income	4	1	4	
Low-middle income	1	4	3	
Upper-middle income	1	0	1	
Annual rainfall (mm)				0.280
Low (< 500)	3	2	2	
Moderate (500–1500)	3	3	3	
High (> 1500)	0	0	3	
Climate				0.404
Tropical humid	3	1	6	
Arid	2	2	1	
Mixt	1	2	1	
Average elevation (m)				0.293
< 800	2	3	6	
≥ 800	4	2	2	

### Factors associated with *T. gondii* seroprevalence

The meta-analysis was performed on 38 of the 60 included articles with eight identified potential predictor variables. The results show that pregnant women who own a cat (OR = 1.584, 95%CI: 1.288–1.948) or consume raw meat (OR = 1.741, 95%CI:1.176–1.842) or raw vegetables (OR = 1.476, 95%CI: 1.079–2.018) were more likely to be seropositive for *Toxoplasma*, with an overall effect, P < 0.05 (Table 5).

**Table 5:** Factors associated with seroprevalence of toxoplasmosis among pregnant women in Africa.

Variables	*Toxoplasma* seropositive		OR (95%CI)	Overall effect (P-value)	Number of studies	References (p<0.05)	References (p>0.05)
	Yes, n (%)	No, n (%)					
**Residence (n = 6,594)**				0.839	22	22, 28, 29, 37, 48, 56, 63	13, 17, 21, 23, 25, 27, 32, 35, 37, 46, 47, 57, 59, 64
Rural	1173 (44.1)	1488 (55.9)	1.03 (0.767–1.387)				
Urban	1966 (50.0)	1967 (50.0)	1				
**Cat ownership (n = 11,555)**				< 0.001	35	25, 26, 28, 30, 37, 40, 45, 48, 54, 56, 59, 65	9, 10, 13, 17, 18, 21, 23, 24, 27, 29, 31, 32, 36, 37, 41, 43, 47, 57, 58, 63, 64, 68
Yes	1739 (52.3)	1586 (47.7)	1.583 (1.272–1.969)				
No	3446 (41.9)	4784 (58.1)	1				
**Consumption of raw meat (n = 10,761)**				0.001	32	15, 26, 31, 40, 43, 47, 48, 54	9, 10, 17, 18, 23–25, 27–30, 32, 35–37, 41, 46, 57–59, 63, 65, 68
Yes	1970 (46.5)	2264 (53.5)	1.503 (1.193–1.895)				
No	3002 (46.0)	3525 (54.00)	1				
**Consumption of raw milk (n = 3,260)**				0.147	9	32, 64	9, 15, 27, 28, 41, 47, 59
Yes	764 (56.4)	591 (43.6)	1.510 (0.866–2.636)				
No	669 (35.1)	1236 (64.9)	1				
**Consumption of raw vegetables (n = 7,812)**				0.015	23	15, 28, 35, 37, 47, 48, 55, 59	9, 10, 13, 17, 18, 24, 25, 27, 30–32, 41, 46, 54, 58
Yes	2361 (50.2)	2346 (49.8)	1.476 (1.079–2.018)				
No	1474 (45.5)	1769 (54.5)					
**Contact with soil (n = 49,999)**				0.142	16	15, 37, 40, 64	13, 18, 23–27, 30, 47, 56, 58, 65
Yes	1610 (70.3)	681 (29.6)	1.258 (0.926–1.710)				
No	1509 (51.0)	1449 (49.0)					
**Blood transfusion (n = 3,936)**				0.367	12	35	9, 10, 17, 23, 25, 27, 28, 37, 47, 54, 64
Yes	118 (53.4)	103 (46.6)	1.244 (0.774–2.001)				
No	1838 (49.5)	1877 (50.5)					
**HIV status (n = 3,351)**				0.086	12	30, 35	31, 10, 23, 24, 37, 43, 46, 48, 57, 59
Positive	149 (52.5)	1135 (47.5)	1.547 (0.941–2.543)				
Negative	1422 (46.4)	1645 (53.6)					

## Discussion

Africa is a continent with a multicultural community that has highly varying living conditions and feeding habits, a high prevalence of communicable diseases, including HIV/AIDS, and most often a low and traditional agriculture-dependent economy level, which can favor the seroprevalence of toxoplasmosis. This systematic review and meta-analysis is the first to assess the predictive factors for *T*. *gondii* chronic infection among pregnant women in Africa. We did not report the pooled prevalence of acute infection because of the unavailability of clearly stated data (IgM or IgG avidity results) in most of the included studies. Nonetheless, the estimation of the prevalence of chronic infection is a widely recongnized indicator of parasite circulation in a given population.

We observed a 42% pooled seroprevalence of *T*. *gondii* infection. The I^2^ statistics results showed the presence of heterogeneity and Egger’s test and trim analysis revealed the presence of publication bias. However, based on the sensitivity analysis, the value for the pooled seroprevalence is robust and high, suggesting a potentially high impact on pregnancy outcomes due to the possible transmission of infection to the fetus. A high level of congenital toxoplasmosis has already been reported for Northern Africa [74–75] but it is still widely unrecognized in sub-Saharan Africa. Recent epidemiological studies reported recombinant and atypical *Toxoplasma* genotypes in severe cases of congenital toxoplasmosis in Tunisia [76–77], as well as in breeding animals [78], showing the circulation of virulent genotypes. Little is known about the distribution of circulating genotypes in sub-Saharan Africa and their virulence. To date, a virulent genotype, Africa-1, has been identified in a few immunocompetent travelers returning to France from Senegal, the Ivory Cost, and the Republic of Congo presenting with fever, myalgia, and neurological signs [79]. These observations suggest that severe congenital cases are likely to occur in sub-Saharan Africa. Knowledge acquired from South America showed that virulent atypical strains can be responsible for fetal loss at any stage of pregnancy and for severe sequelae [80]. In Africa, the presence of virulent genotypes may also be associated with a high burden of disease. In the absence of documentation, these cases are probably not diagnosed. A major study by Mercier et al. [81] showed that nearly half of *Toxoplasma* strains isolated from domestic animals in Gabon were identified as Africa-1 or Africa-3 genotypes, both highly virulent in mice, with a lethal outcome in > 90% of animals. Their phylogenetic analysis concluded that African and American strains share a common ancestor. Thus, given the high observed pooled seroprevalence, it would be of great interest to better evaluate the burden of toxoplasmosis in humans in Africa.

Similar seroprevalence estimates have been reported in systematic reviews conducted on pregnant women in Iran (43%) [82], the general population in Iran (39.3%) [83], and pregnant women who had a history of abortion worldwide (43%) [84]. A similar pooled seroprevalence estimate of toxoplasmosis has also been reported in a systematic review and meta-analysis conducted on HIV-infected pregnant women worldwide (45.7%) [85]. Noteworthy, in this latter study, 10 out of the 14 included studies originated from African countries, which could explain the very similar prevalence, as that observed in our study on non-immunocompromised women. Indeed, the immune background, particularly HIV infection in not known to influence the seroprevalence rate [86].

Our finding is higher than the overall worldwide prevalence of toxoplasmosis among pregnant women (33.8%, 95%CI: 31.5% - 35.9%) [87]. In particular, it is higher than prevalence rates reported in Asia (28.5% in Saudi Arabia [88], 29.9% in Sri Lanka [89], 10% in Japan [90]) and Europe (31% in France [7] and 9% in Norway [91]). Several factors, such as the climate, standard of living, quality of the drinking water, cooking habits of meat, and density of felids, could account for these differences in seroprevalence. Few data are available regarding the prevalence of toxoplasmosis in felids in Africa, and even fewer in wild felids, compared to Europe or America. However, two recent meta-analyses [92,93] reported that the highest *T*.*gondii* pooled seroprevalences in domestic cats worldwide were observed in Australia (52% and 66.6%, respectively) and in Africa (51% and 55.7%, respectively). Interestingly, the highest rate of seroprevalence in wild felids was observed in lions (87.6%) [93]. Besides, the highest rate of *Toxoplasma* oocysts detection in feces from domestic cats (9.8%) was reported from African studies [93]. Although these data need to be consolidated, they indicate that the high prevalence in African felids could support the overall high seroprevalence observed in pregnant women on this continent.

We observed that the Central and East African regions had the highest pooled *T*. *gondii* seroprevalence, whereas the lowest was reported in the South African region. Moreover, the sub-group analysis conducted by country showed four countries from Central and East Africa (Congo, Gabon, Ethiopia, and Eritrea) to be among the top five countries with the highest pooled seroprevalence. The possible reason for the highest seroprevalence in Central and East Africa may be due to the frequency of contact with domestic or stray cats or other felids, and poor environmental hygiene [94]. Geoclimatic conditions in Central Africa, which has a tropical warm humid climate and small temperature range, may also favor long-term survival of oocysts in the environment, as observed in South America, where a high seroprevalence has been documented [95]. Some authors have also postulated that climate could affect the life cycle and population dynamics of prey species, thereby providing favorable conditions for cat reproduction and increasing numbers of young, naive definitive hosts susceptible to *T*. *gondii* infection [96].

The serological test used to diagnose *T*. *gondii* infection in the included studies was recorded, as this could introduce bias depending on its performance [97]. Indeed, latex agglutination is known to yield a certain number of false positive results, whereas the sensitivity of certain RDTs can be lower than that of EIA, as already observed in field studies [98]. However, the prevalence estimates obtained in Ethiopian studies using AT and EIA did not significantly differ (median seroprevalence of 80.55% and 69.60%, respectively, p = 0.74). Interestingly, the sole two studies using RDTs, performed in Nigeria and Zambia, showed the lowest prevalence rates (11.7% and 5.8%, respectively) [22,43] observed in central African countries. Both countries have a humid tropical climate and a low or lower-middle income. *Toxoplasma* seroprevalence was, thus, expected to be high. The study performed in Nigeria by Akubuilo et al. [22] contrasted with other Nigerian studies using EIA, as Dairo et al. [37], Nasir et al. [57], and Oboro et al. [52] reported a seroprevalence of 35.9%, 48.9%, and 65.6%, respectively. This supports the hypothesis of poor sensitivity of the RDT used by Akubuilo et al. [22]. The RDT used in the study performed in Zambia [43] showed poor efficiency in a study comparing three RDTs for *Toxoplasma* diagnosis [99], which could also explain such an unexpectedly low seroprevalence. However, no other data are available from Zambia for other patient groups, and thus these data still need to be consolidated.

In this systematic review and meta-analysis, we confirmed that pregnant women owning a cat had higher odds of seropositivity for toxoplasmosis than those who did not, as observed in previous individual studies from various countries [100–104], including Ethiopia [105,106]. Following primary infection, young cats can spread several hundred thousand oocysts per day for three weeks, and thus play a crucial role in *T*. *gondii* transmission through direct contact or the handling of litter. In addition, stray cats also massively contaminate the environment, water, and vegetables [1,2]. Although experimental infection of cats has shown that re-infection with a same genotype strain did not lead to re-shedding of oocysts in feces, infection with other genotypes did. In Africa, cats are mostly free-ranging or feral animals, thus are likely to encounter multiple genotypes of *T*. *gondii* via hunting various infected preys. The same applies even more likely to wild felids, ensuring multiple re-infections and re-sheeding of oocysts in the environement [96].

Another important risk factor was the consumption of raw meat, which is currently practiced in Eastern Africa. A similar finding was reported in studies conducted in Saudi Arabia, the Philippines, and Brazil [107–109]. Consistent with this finding, a systematic review and meta-analysis of case-controlled studies showed that the consumption of raw meat is a risk factor significantly associated with acute *T*. *gondii* infection in humans (OR = 3.44 (1.29–9.16) [110]. In other studies, not only eating raw meat but also contact with raw meat was shown to be associated with a higher risk of acquiring *Toxoplasma* infection [106].

In accordance with the results of previous studies from other continents [104,109,111–113], we found that the consumption of raw vegetables had a significant overall effect on *Toxoplasma gondii* seropositivity among pregnant women, who had a 47.6% higher risk of acquiring *T*.*gondii* infection. However, the risk associated with raw vegetable consumption was not unanimously recognized, as observed in Iran [82,104], as well as in African countries (9, 10, 14, 18, 19, 24, 26, 28, 31–33, 42, 47, 55, 59). This discrepancy could be due to the type of agriculture, the hygiene of the food market and food handling, the washing habits of vegetables and quality of the water supply, soil contamination by felids, and the density of cats.

This study confirms that the seroprevalence of toxoplasmosis in Africa is high. Recent epidemiological data from animals showed that the Africa-1 genotype predominates in Benin [114] and Gabon [81], meaning that similar strains are likely to infect humans and could be responsible for congenital infection. A retrospective study conducted in Benin on pregnant women estimated that the *Toxoplasma* seroconversion rate was 3.4% [16], which is more than 10-fold higher than that estimated among French pregnant women [7]. Further studies should be undertaken in African countries to evaluate the rate of infected newborns, for example by serological screening at birth, as already carried out in Colombia [115]. The incidence and burden of toxoplasmosis in African countries is currently unknown, whether in the setting of pregnancy or among HIV-infected patients, as biological confirmation is rarely obtained [116]. A call for raising awareness concerning toxoplasmosis and fungal diseases was made several years ago [117], but actions are still pending to better diagnose toxoplasmosis and characterize its true burden. If a high incidence rate is confirmed, serological screening and counseling of all pregnant women for toxoplasmosis infection as a part of antenatal care would be crucial to minimize congenital transmission and its complications.

We were unable to identify new predictors for acquiring toxoplasmosis infection other than those already reported, i.e., contact with cats and the consumption of raw meat and unwashed vegetables. Therefore, efforts should be made towards prevention programs targeting women of child-bearing age, raising awareness of the clinical signs and possible consequences for the fetus, and focusing on hygienic prevention measures to avoid infection, such as the proper handling and cooking of meat, washing of vegetables and hands, and management of domestic cats [2]. We believe that our study should pave the way for epidemiological studies on humans and encourage policymakers to fund prevention programs.

## Supporting information

S1 File Searching strategy. (DOCX)

S2 File Measure of the true agreement (K) between the raters. (DOCX)

S3 File The revised 2023 JBI checklist for analytical cross-sectional studies to assess the quality ranking of the full extracted articles. (DOCX)

S4 File Sensitivity analysis of the pooled prevalence of *T*. *gondii* infection among pregnant women in Africa for a single study omitted. (DOCX)

S5 File Database. (XLSX)
