# Supplementary material for: Toxoplasma gondii seroprevalence among pregnant women in Africa: A systematic review and meta-analysis
Source: PLoS Negl Trop Dis. 2024 May 23;18(5):e0012198. doi: 10.1371/journal.pntd.0012198 (PMC11152313; doi:10.1371/journal.pntd.0012198)
Supplement: S1 File — (DOCX) [file pntd.0012198.s001.docx]

| Search | Query | Items retrieved |
| --- | --- | --- |
| PubMed/Medline | (("Toxoplasmosis"[Mesh]) OR ("Toxoplasma"[Mesh])) AND ("Pregnant Women"[Mesh]) AND ("Africa"[Mesh]) | 11 |
| From 2010/01/01 to 2023/03/30 | (((toxoplasmosis[Title/Abstract]) OR (toxoplasma[Title/Abstract])) AND (pregnant women[Title/Abstract])) AND (africa[Title/Abstract]) | 27 |
|  | …AND (ethiopia[Title/Abstract]) | 19 |
|  | …AND (nigeria[Title/Abstract]) | 11 |
|  | …AND *every African country* | … |
|  | … |  |
| PubMed/Medline using All fields | ((Toxoplasmosis) OR (toxoplasma)) AND (pregnant women) AND Africa | 99 |
| Europe PMC | (prevalence of toxoplasmosis infection among pregnant women in Africa AND (((SRC:MED OR SRC:PMC OR SRC:AGR OR SRC:CBA) NOT (PUB_TYPE:"Review"))) AND (FIRST_PDATE:[2010 TO 2023]) | 488 |
| PMC; US national library of Medicine National institute of health | (("toxoplasmosis"[MeSH Terms] OR "toxoplasmosis"[All Fields]) OR ("toxoplasma"[MeSH Terms] OR "toxoplasma"[All Fields])) AND (("pregnant women"[MeSH Terms]) OR ("pregnant"[All Fields] AND "women"[All Fields]) OR "pregnant women"[All Fields])) AND ("africa"[MeSH Terms] OR "africa"[All Fields]) AND ("2010/01/01"[PubDate] : "2023/03/31"[PubDate]) | 1713 |
| AJOL | seroprevalence of "Toxoplasma gondii infection" among "pregnant women" in "Africa" | 47 |

**^S1 File : Searching strategy^**
