# Supplementary material for: Toxoplasma gondii seroprevalence among pregnant women in Africa: A systematic review and meta-analysis
Source: PLoS Negl Trop Dis. 2024 May 23;18(5):e0012198. doi: 10.1371/journal.pntd.0012198 (PMC11152313; doi:10.1371/journal.pntd.0012198)
Supplement: S2 File — (DOCX) [file pntd.0012198.s002.docx]

|  |  | Yared Mulu Gelaw (R2) | | Total |
| --- | --- | --- | --- | --- |
| Gizachew Worku Dagnew (R1) |  | Relevant | Not-relevant |  |
|  | Relevant | 1. 60 | 1. 2 | (g1) 62 |
|  | Not relevant | (c) 3 | (d) 17 | (g2) 20 |
| Total | | (f1) 63 | (f2) 19 | (n) 82 |

**S2 File : Measuring the true agreement (K) between the raters** [1]

- The true agreement (K) between the rater (k$\text{)}=\frac{observed agreement\left( Po \right)-chance agreement (Pc)}{1-chance agreement (pc)}$
- Observed agreement among raters (Po$\text{)}=\frac{a+d}{n}$

(Po$\text{)}=\frac{60+17}{82}$=0.94

- Chance agreement $(PC)= \frac{(\frac{f1*g1}{n})+(\frac{f2*g2}{n})}{n}$

$\left( PC \right)= \frac{\left( \frac{63*62}{82} \right)+\left( \frac{19*20}{82} \right)}{82}=0.637$

- The true agreement (K) between the rater (k$\text{)}=\frac{\left( Po \right)-(Pc)}{1- (pc)}$

(k$\text{)}=\frac{0.94-0.637}{1- 0.637}=\boldsymbol{0.832}$

Interpreting the Magnitude of Kappa

The result of the kappa coefficient based on the Landis and Koch [2] proposed standards for strength of agreement interpreted as follows;

- 0 poor,
- 0.01–0.20 slight,
- 0.21–0.40 fair,
- 0.41–0.60 moderate,
- 0.61–0.80 substantial, and
- **0.81–1 almost perfect agreement between raters, and our (K) coefficient was 0.832.**

Reference

[1] J. Sim and C. C. Wright, “The kappa statistic in reliability studies: use, interpretation, and sample size requirements,” *Phys. Ther.*, vol. 85, no. 3, pp. 257–268, 2005.

[2] J. R. Landis and G. G. Koch, “The measurement of observer agreement for categorical data,” *biometrics*, pp. 159–174, 1977.
