# Supplementary material for: Toxoplasma gondii seroprevalence among pregnant women in Africa: A systematic review and meta-analysis
Source: PLoS Negl Trop Dis. 2024 May 23;18(5):e0012198. doi: 10.1371/journal.pntd.0012198 (PMC11152313; doi:10.1371/journal.pntd.0012198)
Supplement: S3 File — (DOCX) [file pntd.0012198.s003.docx]

**S3 File: The revised 2023 JBI checklist for analytical cross-sectional studies to assess the quality ranking of the full extracted articles**

| S. no. | Question | Yes/No |
| --- | --- | --- |
| 1 | Were the criteria for inclusion in the sample clearly defined? |  |
| 2 | Were the study subjects and the setting described in detail? |  |
| 3 | Was the exposure measured in a valid and reliable way? |  |
| 4 | Were objective, standard criteria used for measurement of the condition? |  |
| 5 | Were confounding factors identified? |  |
| 6 | Were strategies to deal with confounding factors stated? |  |
| 7 | Were the outcomes measured in a valid and reliable way? |  |
| 8 | Was appropriate statistical analysis used? |  |
|  | Total Yes |  |
