# Supplementary material for: Toxoplasma gondii seroprevalence among pregnant women in Africa: A systematic review and meta-analysis
Source: PLoS Negl Trop Dis. 2024 May 23;18(5):e0012198. doi: 10.1371/journal.pntd.0012198 (PMC11152313; doi:10.1371/journal.pntd.0012198)
Supplement: S4 File — (DOCX) [file pntd.0012198.s004.docx]

**S4 File: Sensitivity analysis of the pooled prevalence of T. gondii infection among pregnant women in Africa for a single study omitted**

| Study omitted | Ref no | Estimate | [95% Conf. Interval] |
| --- | --- | --- | --- |
| Okojokwu O et al. (2023) | 15 | 43.14 | 36.93, 49.35 |
| Hassan SA et al. (2023) | 14 | 42.85 | 36.63, 49.07 |
| Lushina M et al. (2022) | 17 | 43.16 | 36.93, 49.38 |
| Dambrun M et al. (2022) | 16 | 42.72 | 36.48, 48.96 |
| Singh B et al. (2021) | 20 | 42.64 | 36.44, 48.85 |
| Adugna B et al. (2021) | 19 | 42.41 | 36.26, 48.57 |
| Laboudi M et al. (2021) | 18 | 42.89 | 36.64, 49.13 |
| Abdelbaset AE et al. (2020) | 28 | 43.22 | 37.02, 49.42 |
| Nguemaïm NF et al. (2020) | 27 | 43.02 | 36.82, 49.23 |
| Tecle AS et al. (2020) | 26 | 42.71 | 36.51, 48.91 |
| Mulugeta et al. (2020) | 25 | 42.46 | 36.29, 48.65 |
| Zakari M et al. (2020) | 24 | 43.13 | 36.91, 49.35 |
| Van der Colf BE et al. (2020) | 9 | 43.58 | 37.73, 49.42 |
| Akubuilo AS et al. (2020) | 22 | 43.42 | 37.23, 49.61 |
| Khames M et al. (2020) | 72 | 43.20 | 36.92, 49.47 |
| Hoummadi L et al. (2020) | 21 | 43.13 | 36.49, 49.77 |
| Todjom FG et al. (2019) | 32 | 42.84 | 36.64, 49.05 |
| Mirambo MM et al. (2019) | 31 | 43.10 | 36.88, 49.32 |
| Teweldemedihin et al. (2019) | 30 | 43.01 | 36.79, 49.24 |
| Fenta DA (2019) | 29 | 42.22 | 36.20, 48.24 |
| Paul E et al. (2018) | 39 | 42.86 | 36.65, 49.07 |
| Mohammed WM et al. (2018) | 38 | 43.23 | 37.02, 49.45 |
| Dairo MD et al. (2018) | 37 | 43.01 | 36.78, 49.24 |
| Hafez Hassanain et al. (2018) | 73 | 43.27 | 37.05, 49.49 |
| Adeniyi OT et al. (2018) | 35 | 43.10 | 36.87, 49.33 |
| Jula J et al. (2018) | 34 | 43.21 | 36.98, 49.44 |
| Mabeku LB et al. (2018) | 33 | 43.01 | 36.76, 49.26 |
| Völker F et al. (2017) | 51 | 42.38 | 36.21, 48.55 |
| Negero J et al. (2017) | 50 | 42.33 | 36.18, 48.49 |
| Yohanes T et al. (2017) | 49 | 42.27 | 36.14, 48.41 |
| Negussie A et al. (2017) | 48 | 43.02 | 36.80, 49.24 |
| Bamba S et al. (2017) | 47 | 43.10 | 36.87, 49.31 |
| Saajan AM et al. (2017) | 46 | 42.86 | 36.64, 49.08 |
| Murebwayire E et al. (2017) | 45 | 43.41 | 37.22, 49.60 |
| Ballah F et al. (2017) | 44 | 43.14 | 36.91, 49.37 |
| Frimpong C et al. (2017) | 43 | 43.52 | 37.44, 49.61 |
| El-Shqanqery HE et al. (2017) | 42 | 43.11 | 36.85, 49.36 |
| Pegha Moukandja I et al. (2017) | 41 | 42.64 | 36.43, 48.86 |
| Tlamcani Z et al. (2017) | 40 | 42.94 | 36.47, 49.42 |
| Kwofie KD et al. (2016) | 55 | 42.97 | 36.77, 49.17 |
| Ayi I et al. (2016) | 54 | 42.75 | 36.55, 48.95 |
| Abamecha F et al. (2016) | 53 | 42.17 | 36.09, 48.25 |
| Oboro IL et al. (2016) | 52 | 42.50 | 36.32, 48.69 |
| Elichilia R et al. (2015) | 59 | 42.91 | 36.70, 49.11 |
| Agmas B et al. (2015) | 58 | 42.46 | 36.28, 48.63 |
| Gelaye W et al. (2015) | 10 | 42.16 | 36.11, 48.21 |
| Nasir IA et al. (2015) | 57 | 42.79 | 36.57, 49.00 |
| Awoke K et al. (2015) | 56 | 43.30 | 37.09, 49.52 |
| Oyinloye SO et al. (2014) | 64 | 43.23 | 37.03, 49.43 |
| Abdel-Raouff M et al. (2014) | 63 | 43.27 | 37.07, 49.47 |
| Bamba S et al. (2014) | 62 | 43.03 | 36.80, 49.25 |
| Endris M et al. (2014) | 61 | 42.11 | 36.20, 48.01 |
| Doudou Y et al. (2014) | 60 | 42.25 | 36.29, 48.20 |
| Mwambe B et al. (2013) | 65 | 43.09 | 36.87, 49.32 |
| Linguissi LS et al. (2012) | 68 | 43.27 | 37.06, 49.48 |
| Zemene E et al. (2012) | 67 | 42.20 | 36.09, 48.31 |
| El Deeb HK et al. (2012) | 66 | 42.47 | 36.30, 48.65 |
| Njunda AL et al. (2011) | 70 | 42.44 | 36.25, 48.62 |
| Sattti AB et al. (2011) | 69 | 43.35 | 37.14, 49.56 |
| Sitoe SP et al. (2010) | 71 | 43.30 | 37.09, 49.50 |
